# Plasma Levels of Polychlorinated Biphenyls, Non-Hodgkin Lymphoma, and Causation

**DOI:** 10.1155/2012/258981

**Published:** 2012-04-03

**Authors:** Michael D. Freeman, Sean S. Kohles

**Affiliations:** ^1^Department of Public Health and Preventive Medicine, Oregon Health & Science University School of Medicine, 1234 SW 18th Avenue, Suite 102, Portland, OR 97205, USA; ^2^Regenerative Bioengineering Laboratory, Department of Mechanical & Materials Engineering, Portland State University, 1930 SW 4th Avenue, Suite 400, Portland, OR 97201, USA

## Abstract

Polychlorinated biphenyls (PCBs) are synthetic chlorinated hydrocarbons that have extensively polluted the environment and bioaccumulated in the food chain. PCBs have been deemed to be probable carcinogens by the Environmental Protection Agency, and exposure to high levels of PCBs has been consistently linked to increased risk of non-Hodgkin lymphoma (NHL). In the present article we present a forensic epidemiologic evaluation of the causal relationship between NHL and elevated PCB levels via application of the Bradford-Hill criteria. Included in the evaluation is a meta-analysis of the results of previously published case-control studies in order to assess the strength of association between NHL and PCBs, resulting in an odds ratio in which the lowest percentile PCB concentration (quartile, quintile, or tertile) has been compared with the highest percentile concentration in the study groups. The weight-adjusted odds ratio for all PCB congeners was 1.43 with a 95% confidence interval of 1.31 to 1.55, indicating a statistically significant causal association with NHL. Because of the lack of an unexposed comparison group, a rationale for the use of a less than 2.0 relative risk causal contribution threshold is presented herein, including an ecologic analysis of NHL incidence and PCB accumulation (as measured by sales volume) over time. The overall results presented here indicate a strong general causal association between NHL and PCB exposure.

## 1. Introduction

Polychlorinated biphenyls (PCBs) are a class of commercially-produced organochlorines known as chlorinated hydrocarbons [1]. PCBs are nonflammable, chemically stable, have a high boiling point, and are nearly insoluble in water. In addition, they are resistant to the effects of oxidation, acids, bases, and other chemicals [2, 3]. A PCB molecule consists of a pair of joined 6-carbon rings, with chlorine(s) attached or “substituted” at any one of the free 10-carbon positions. There are 209 possible chlorine arrangements, which are called congeners. It is the number of the chlorines and where they are attached that determine the congener's properties. A congener's two-carbon rings can be twisted, relative to each other, or they can be aligned in the same plane (coplanar). Coplanar carbon ring alignment occurs when the chlorine(s) are attached to the carbons closest to the link between the two rings, called the ortho position. Coplanar congeners exhibit dioxin-like properties [4, 5].

For approximately 50 years (beginning in 1929), PCBs were manufactured in the United States (USA) and were used in numerous industrial and commercial applications in the form of mixtures called Aroclors [6]. One manufacturer (Monsanto Company) has produced ~99% of all the PCBs used in the USA [2]. Worldwide, Monsanto has produced between 39 and 48 percent of all PCBs. Widespread industrial use of PCBs, combined with improper disposal practices, has led to the introduction of PCBs into the environment, where these chemicals are found in all environmental media, including air, water, and soil. Based on mounting evidence that PCBs were accumulating and persisting in the environment and that this accumulation was causing adverse health effects in humans and animals, PCB production in the USA was halted in the late 1970s [1].

 PCBs are highly soluble in lipids, and as such, are absorbed by fish and other animals, leading to considerable bioaccumulation in the food chain. PCBs are typically absorbed into the human body through ingestion, inhalation, and/or dermal exposure [1, 7–9]. Bioaccumulation in humans and animals occurs when PCBs are absorbed into the body at a rate greater than the rate at which they are metabolized and excreted from the body. Detectable levels of PCBs have been found in humans in adipose (fat) tissue, breast milk, hair, and serum lipids [3, 10]. Consumption of contaminated food is the major source of PCB exposure for the general United States population that is not occupationally exposed to PCBs [1, 2, 8, 10, 11]. Unsurprisingly, populations that are at particular risk to high exposure to PCBs include people who consume sport-caught fish and other contaminated foods, workers occupationally exposed to PCBs, individuals residing near hazardous waste sites containing PCBs, and nursing infants [7, 8, 10, 12].

 PCBs are deemed to be probable carcinogens by the Environmental Protection Agency based on the results of both animal and human studies [1]. A number of animal studies have demonstrated a direct dose-response relationship between PCB levels and liver tumor occurrence [13–16]. In humans, there is also good evidence of an association between increased cancer rates and PCBs. A number of epidemiologic studies have indicated increased rates of liver and biliary cancer [17], breast cancer [18], skin cancer [19], and Non-Hodgkin Lymphoma (NHL), among other cancers [20].

 PCBs appear to have a number of toxic qualities that potentially explain these observed associations, including dioxin-like characteristics of some congeners (118, 156, and 169) [5], the ability to mimic hormones for others [11], and neuro- and immunotoxicity as well [21–23]. This last quality (immunotoxicity) is helpful in explaining the observed association between elevation of certain congener titers (118, 138, 153, 170, and 180) and the increased incidence of NHL, as immune system depression is considered to be one of the strongest risk factors for NHL [24, 25]. Immunotoxicity is a characteristic shared by other organochlorines as well, including dioxins and chemicals found in pesticides and herbicides such as hexachlorobenzene, heptachlor, chlordane, and others [26], which have also been found to be associated with increased risk of NHL [27]. It is most likely the quality of immunotoxicity that links both PCBs and non-PCB organochlorines to increased incidence of NHL, and when both are present, each contribute to the risk of NHL independently [26].

 NHL includes all cancers of lymphoid tissues except Hodgkin's disease, a malignancy of the lymph nodes [28]. The incidence of NHL in many parts of the world is rising more rapidly than the incidence of virtually all other human cancers [29, 30]. In the US, the incidence of NHL increased at an average of 3.6% per year from 1975–1991 and continued to rise over the period 1991–2005, albeit at a slower rate (~0.5% annually) [31, 32]. It is widely accepted that changes in diagnostic practices and known risk factors such as age, autoimmune disease incidence, and prevalence of immune-suppressing infections are insufficient to explain the emerging “epidemic” of NHL observed in most of the world [33, 34]. Additionally, it is hypothesized that exposures shared by many populations worldwide are the most likely explanation for the steep increases in NHL incidence [31, 33–35], and that this exposure is likely immunotoxic or immunosuppressive [36]. Thus, exposure to PCBs and other persistent organic pollutants provides a reasonably plausible explanation, at least in part, for the rise in NHL incidence during the latter half of the 20th century.

 The underlying physiologic mechanisms for the development of NHL secondary to PCB exposure are not fully known. It is well established that the dioxin-like congeners can bind to and activate the aryl hydrocarbon receptor (AhR), a normally inactive transcription factor [37]. The overactivation of the AhR can induce enzymes that produce cytotoxic metabolites or otherwise adversely affect cellular metabolism [38]. The degree to which a particular coplanar dioxin-like congener can act on the AhR is measured in Toxic Equivalents (TEQ), a comparison with a standard set to the highly toxic dioxin-like compound 2,3,7,8-Tetrachlorodibenzo*-p-*dioxin (TCDD) [39]. Less is known about the noncoplanar nondioxin-like congeners, however, they appear to affect immune function by direct cellular effects, including inhibition of leukocytic phagocytosis, among other actions [22].

 Although there are some differences in the specific congeners measured and variations in the PCB levels associated with increased risk of NHL, the literature generally supports, via both case-control and cohort studies, the concept that populations with higher levels of exposure to certain PCB congener and with higher body burdens of these same congeners are at increased risk for NHL. Multiple representative publications describe groups of previously identified cases of NHL from various registries and clinical investigations and compared them with age and gender-matched controls, resulting in quantitative descriptions of relative influence of percentile groupings of blood plasma PCB on NHL risk via conditional logistic regression (Table 1) [26, 40–48]. An additional study [49], analyzed adipose tissue in a postmortem assessment. Rothman et al. [40], Engel et al. [41, 48], all used prediagnosis blood samples (not subject to weight loss bias). Additionally, three landmark exposure studies investigated the association between environmental levels of various Aroclor products and mortality rates from NHL (Table 2) [50–52]. These studies collectively have provided a foundation for a reasonable conclusion that a causal relationship exists between high levels of PCBs, both in the environment and in the body and increased risk of NHL.

A few authors have reported subgroups of NHL that are linked to PCB exposure, including an association between diffuse large cell lymphoma and congener 118 and T-cell lymphoma and congener 180 [42]. Hardell reported the highest observed association in their cohort between elevated PCB burdens and low-grade B-cell lymphomas, the most prevalent category of NHL subtypes [43]. DeRoos found an association between congeners 180 and 187 and diffuse but not follicular NHL, whereas Morton reported an association between follicular NHL and congener 180, as well as marginal zone NHL subtype [54].

Given a reasonable basis for a conclusion of general (population-based) causation, the question arises of how to determine, in an individual case of NHL in which elevated blood and/or adipose levels of total or individual PCB congeners have been observed, if the relationship between the two is causal. Far more has been written in epidemiology about the evaluation of general (population) causation than specific (individual) causation. Epidemiologic methods are used for the investigation of specific causation in both short acting exposure/outcome situations (i.e., outbreak/injury investigation) and in settings in which disease onset is latent, sometimes for decades, following exposure, such as with exposure to environmental toxicological or radiological hazards. The discipline of forensic epidemiology is directed at addressing, often for presentation in legal settings, the specific causal correlation between a suspected hazard and a disease or injury outcome [55–57]. The ultimate goal of the investigation is to answer the “but-for” question, which is “*but-for the exposure to the hazard, would the individual still have the disease or injury?*” [58]. The forensic epidemiologic approach to answering this question is accomplished by first assessing the plausibility of a causal relationship via application of the Bradford-Hill criteria and then estimating the attributable risk percent or probability of causation (PC) that quantifies the probability that, but for the exposure, the disease or injury outcome would not have occurred [59]. Generally, if the PC exceeds 50% then the hazardous exposure is considered most probably causally related to the disease or injury outcome [60]. PC is derived from comparative epidemiologic data that adequately represent the relative risk (RR) of disease or injury between *hazard-exposed* populations most meaningfully similar to the individual versus *nonhazard-exposed* populations most meaningfully similar to the individual. A PC of >50% is the equivalent of an RR >2.0 and implies that the cause of disease or injury in an individual randomly selected from the exposed population is the hazard of interest more often than not. This approach is relatively straightforward for evaluating the causal relationship between, for example, cigarette smoking and pancreatic cancer, as there are clearly defined and easily identified exposed (smoking) and unexposed (nonsmoking, not exposed to second-hand smoke) populations.

For the evaluation of PCB exposure and NHL, the issue of causation is more complicated, as the ubiquitous nature of PCBs in the environment means that there are no truly unexposed populations. In combination with the likely multifactorial etiology of NHL (viral infections, immune system depression, autoimmune conditions, and genetic anomalies are all thought to play a role), it is much easier to conclude that the prominence of a single “suspect” hazard-like elevated serum levels of PCBs serves to *contribute* to the cause of an individual's NHL such that the hypothetical complete subtraction of the hazard would likely result in the individual not developing the disease (the factor is *necessary *for the development of the condition). Conversely, the development of NHL most likely requires a number of components along with the suspect hazard, any number of which may also be necessary in order for the disease to manifest.

 In the present endeavor, we propose a methodologic framework for the evaluation of specific causation of NHL associated with PCB exposure that takes into account the pervasive and persistent nature of PCBs in the environment and the difficulties with setting an absolute threshold for specific causation. To accomplish this task, we present a meta-analysis of the previously published case-control studies that have examined the risk of NHL relative to PCB titer by congener. Additionally, we discuss a rationale for the use of an adjusted relative risk or probability of causation threshold for concluding that a causally contributory relationship is more probably than not present in an individual with NHL and elevated PCB titers.

## 2. Methods

### 2.1. Application of Hill Criteria for Causal Assessment

 For the purposes of the present study, a causal relationship is defined as when an exposure is found to have served as an antecedent event or condition that was necessary for the occurrence of a specific disease or injury at the moment that it occurred, given that other conditions are fixed [61]. That is to say, the cause of the disease or injury is an event or condition that preceded the disease or injury and *without which the disease or injury would not have occurred.* Forensic applications of epidemiology that address the evaluation of causation generally follow the criteria set forth by Sir Austin Bradford-Hill in 1965 [55, 56, 62–66]. Hill's nine criteria, in the order in which they were given in the original publication, are briefly as follows.

1. *Strength of association*: strength of association is the most important determinant of both general and specific causation and quantified by RR, in that the larger the ratio between the incidence of the condition in the exposed group versuses the incidence in the unexposed group, the greater the probability that the relationship is causal. As described previously, an RR >2.0 is the equivalent of a PC >50%, meaning that it is more probable than not that the suspected causal relationship is true.
2. *Consistency*: the repetitive observation of a relationship in different circumstances strengthens the causal inference.
3. *Specificity*: the degree to which a suspected causal factor is associated with a particular outcome or population.
4. *Temporality*: the potential causal factor must precede the outcome it is assumed to affect, and the outcome cannot either occur before it is physiologically feasible or after too great of a latency period. Temporality is the one factor that must always be present in general and specific causation in order to conclude that a cause and effect relationship is present.
5. *Biological gradient*: the injury outcome increases proportionately with increasing dose of exposure (also known as dose-response).
6. *Plausibility*: the degree to which the observed association can be explained by known scientific principles.
7. *Coherence*: a causal conclusion should not fundamentally contradict present substantive knowledge; it should “make sense” given current knowledge.
8. *Experiment*: in some cases, there may be evidence from randomized experiments on animals or humans.
9. *Analogy*: an analogous exposure and outcome may be translatable to the circumstances of a previously unexplored causal investigation.

For application in a forensic setting, these criteria are sometimes modified to include *cessation* (of exposure) as a test-retest criteria and consideration of alternative explanations for the association (such as bias and confounding), in place of the less frequently useful *experiment *criterion [60]. The only criterion that is truly essential for a causal association is *temporality*, as the outcome *must* follow the exposure in time. In fact, none of the criteria (with the exception of *temporality*) are applicable in all circumstances [67, 68].

 The causation criteria can be lumped into three main groups by their utility: (1) those used to evaluate whether there is a reasonably plausible relationship between outcome and exposure (*consistency, specificity, biological gradient, plausibility, coherence, experiment, *and* analogy* and *cessation*); (2) *temporality*; (3) *strength of association* (or probability of causation). An analysis involving the application of these criteria to the evaluation of the plausibility of a causal relationship between NHL and elevated PCB congener titers, as well as temporality, is presented in the results and discussion sections. The final criterion, strength of association, is discussed below.

### 2.2. Meta-Analysis of Individual Congeners as a Measure of Strength of Association (Causal Contribution)

 In order to assess the utility of PCB congener level as an index of causal contribution, it was necessary to meta-analyze data from previously published case-control studies in which the risk of NHL was compared with serum levels of individual congeners through the application of conditional logistic regression. In order to be included in the meta-analysis, the studies needed to have the following characteristics in common: they examined individual PCB congeners identified in plasma samples acquired from cases of NHL (as opposed to groups of congeners), they included age and gender frequency-matched controls, they ranked their results by percentile of congener concentration (from lowest to highest tertile, quartile, or quintile), they quantified their results in terms of natural log odds ratios as a measure of NHL risk by comparing the minimum percentile with the maximum, and multiple strata were available for any included congener. For the meta-analysis, the fixed-effect Mantel-Haenszel (MH)-adjusted odds ratio (OR_MH_) was calculated as a weighted average (*w*_*i*_) of the natural log (ln) odds ratio (OR_*i*_) for each study in which the minimum percentile was compared with the highest percentile concentration (Table 3). This minimum-maximum comparison was chosen for the meta-analysis in order to summarize the comparison of the extrema. The following calculation was used:

(1) $$\text{O}{\text{R}}_{\text{MH}}=\frac{\sum_{i=1}^{k}\left(\left(b_{i}c_{i}/N_{i}\right)\times \left(a_{i}d_{i}/b_{i}c_{i}\right)\right)}{\sum_{i=1}^{k}b_{i}c_{i}/N_{i}}=\frac{\sum_{i=1}^{k}w_{i}\text{O}{\text{R}}_{i}}{\sum_{i=1}^{k}w_{i}}.$$

This approach utilized the subpopulations within each study (*a*_*i*_, *b*_*i*_, *c*_*i*_, and *d*_*i*_) as identified in the two-by-two contingency tables stratified by *k* studies. The confidence interval for the OR_MH_ was determined by first calculating the standard error (SE) of the OR estimate (OR_*i*_) and given by the following equation [69]:

(2) $$\begin{array}{cc} & \text{SE}\left(\ln\text{O}{\text{R}}_{\text{MH}}\right)\\ & \;=\sqrt{\frac{\sum_{i=1}^{k}\left(P_{i}R_{i}\right)}{2{\left(\sum_{i=1}^{k}R_{i}\right)}^{2}}+\frac{\sum_{i=1}^{k}\left(P_{i}w_{i}+Q_{i}R_{i}\right)}{2\left(\sum_{i=1}^{k}R_{i}\right)\left(\sum_{i=1}^{k}w_{i}\right)}+\frac{\sum_{i=1}^{k}\left(Q_{i}w_{i}\right)}{2{\left(\sum_{i=1}^{k}w_{i}\right)}^{2}}}, \end{array}$$

where

(3) $$\begin{array}{c} P_{i}=\frac{a_{i}+d_{i}}{N_{i}},\;\;Q_{i}=\frac{b_{i}+c_{i}}{N_{i}},\\ R_{i}=\frac{a_{i}\times d_{i}}{N_{i}},\;\;w_{i}=\frac{b_{i}\times c_{i}}{N_{i}}. \end{array}$$

The meta-analyzed 95% confidence level (CI) was then calculated in the natural logarithmic (ln) scale to match the scale of the OR:

(4) $$\begin{array}{c} 95\text{\%}\;\text{CI}\left(\ln\text{OR}\right)=\ln\text{OR}\pm \left[1.96\times \text{SE}\left(\ln\text{OR}\right)\right]. \end{array}$$

A test for homogeneity was also conducted to assess the application of the fixed-effect model applied here to the published ORs. This approach was applied to test whether the population ORs are in fact constant across the different strata [70]. If the test fails, the ORs can simply be reported as distinct values or be further meta-analyzed using a random-effects model [27, 71]. The test for homogeneity evaluates the null hypothesis (*H*_*o*_) where the population odds ratios for the *g* tables are assumed statistically identical, or equivalently, *H*_*o*_ = OR_1_ = OR_2_ = ⋯ = OR_*i*_ = ⋯ = OR_*g*_. To perform this test, we calculated the chi-square statistic (*Q*  or  *χ* ^2^):

(5) $$Q=\chi^{2}=\overset{g}{\underset{i=1}{\sum }}w_{i}{\left(y_{i}-Y\right)}^{2}.$$

Here the natural logarithm of each estimated OR is determined:

(6) $$y_{i}=\ln{\text{OR}}_{i}$$

and used to produce a weighted average (*Y*) applying the weighting value described in (1), such that:

(7) $$Y=\frac{\sum_{i=1}^{g}w_{i}y_{i}}{\sum_{i=1}^{g}w_{i}}.$$

The resulting statistic from (5) has a distribution that is approximately chi-square with g-1 degrees of freedom. A chi-square distribution table was then consulted for each test, producing a *P* value as an assessment of the null hypothesis, where *P* < 0.05 was considered as a rejection of the null [70]. An alternative test statistic for assessing homogeneity is the likelihood ratio test, which is computationally more cumbersome than the *Q* statistic applied here [72].

### 2.3. Ecological Analysis

In order to explicate the correlation between changes in environmental levels of PCBs and changes in the incidence of NHL an ecologic effect analysis was performed. Data were acquired from the National Institute of Cancer [32] and compared to PCB production and sales levels [6, 73]. Time-dependent accumulation of PCBs was calculated from the annual sales data of the open source materials including heat transfer products, hydraulics/lubricants, miscellaneous industrial products, plasticizers, and petroleum additives. The environmental PCB accumulation data are presented in three forms: first, only the sales data are plotted over time; second, the sales data are extended in time, assuming a static level of accumulation where the maximum presence of PCB in the environment remains fixed and constant after production ceased; third, the sales data are used as a basis from which to assume a dynamic accumulation of PCB, where environmental levels increase as devices and materials break down, continuously releasing congeners into the environment. This last approach required forecasting the accumulation beyond the end of production following a similar initial growth curve into the future. A polynomial curvefit produced a strong phenomenological model representing the time-dependent accumulation in units of kilo-lbs (*R* ^2^ = 0.9772), with time measured in years:

(8) $$\text{PCB Accumulation}=16,488.2+1048.9\;{\left(\text{Time}\right)}^{2}.$$

This model was then applied to years beyond the end of the sales period, extending the accumulation data into the future, matching the surveillance period for NHL incidence. Plotted comparisons were made over the actual dates (calendar years) as well as relative time from origination (both in sales and cancer incidence monitoring).

The environmental PCB accumulation model was juxtaposed with the incidence over time of a number of major cancer types. A correlation coefficient was calculated for each NHL and non-NHL association, where *R* ^2^ > 0.80 was considered a strong statistical correlation.

## 3. Results

### 3.1. Assessment of the Hill Criteria for Causation

#### 3.1.1. Plausibility

 In the present context, plausibility does not refer to Hill's narrow use of the term as one of the nine causal criteria, in which he referred to the specific biologic action by which an environmental factor caused a disease, but rather to the group of criteria (all but temporality and strength of association) that answer the question “*can the exposure cause the outcome*?” Interestingly, Hill did not consider the biological plausibility criterion to be particularly critical to a finding of cause and effect, stating that he was “*convinced this is a feature that we cannot demand*” [62]. This was because of the recognition that an environmental toxin may, in fact, cause a disease by a currently unexplicated mechanism that may be described in the future.

 In examining the plausibility of a causal relationship between PCB exposure and NHL, there is a substantial amount of published information to rely upon that indicates that the relationship is indeed plausible. Analogy is strongly supported for PCB exposure as a cause of NHL, as they belong to the same chemical family of organochlorines as other chemicals that have been associated with NHL in prior epidemiologic studies [27]. From a biological plausibility perspective, NHL is a disease that has been repeatedly shown to be related to compromise of the body's immune response [24, 25], and there are a number of PCB congeners that have been deemed immunotoxic [74]. Additionally, the dioxin-like PCB congeners have the ability to bind to the aryl hydrocarbon receptor, a normally inactive transcription factor that when bound can alter genetic transcription.

Consistency of the relationship is seen with the number of published epidemiologic studies, both case-control and cohort design, of various populations in various settings in which an association between PCB congener titer levels has been linked to NHL risk (Tables 1 and 2).

#### 3.1.2. Temporality

 Because of the ubiquitous nature of PCBs in the environment and the way in which they accumulate over time in the body, as well as the nature of NHL as a disease that has seen its largest increases in the population over the age of 55, aside from the youngest patients with NHL (often those with readily apparent explanations for the disease, such as immunosuppressive infections), temporality is assumed to be appropriately present in most cases of NHL in the presence of high PCB titers.

#### 3.1.3. Strength of Association (Causal Contribution) via Meta-Analysis

**Figure 1:**
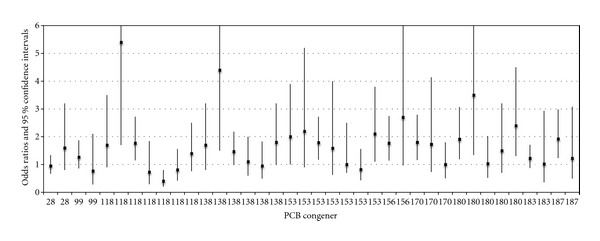
Odds ratios and 95% confidence limits for all PCB blood-level congeners as associated with NHL and reported in six case-control studies.

**Figure 2:**
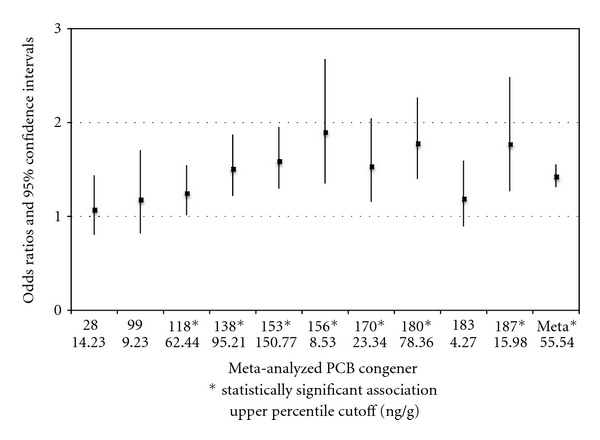
Meta-analyzed congeners indicating Mantel-Haenszel-adjusted odds ratios and 95% confidence limits for all PCB blood-level congeners with two or more strata including the OR_MH_ for all congeners combined. Congeners with single study analyses are also included (congeners 99, 156, 183, and 187). Statistically significant confidence intervals are indicated (∗). Meta-analyzed upper percentile cutoff PCB blood-level values are also included for each congener (ng/g lipid).

Eleven published case-control studies reported on the association between NHL and PCB levels were considered for meta-analysis (Table 1). Of these publications, six articles described seven unique populations associating the influence of 10 congeners (28, 99, 118, 138, 153, 156, 170, 180, 183, and 187) on NHL incidence and which were deemed eligible under the meta-analysis inclusion criteria. Point-estimate odds ratio results for all studied congeners from each of the published case-control studies are described via horizontal forest plot as a summary of the previous published results (Figure 1). These results were then meta-analyzed according to the methods described previously (Figure 2).

The weight-adjusted odds ratio (OR_MH_) for all 10 congeners collectively was 1.43 (95% CI 1.31–1.55). Each of the 10 congeners contributed to its own congener-specific meta-analysis as well as toward the all-congener OR_MH_. OR_MH_ results for seven congeners (118, 138, 153, 156, 170, 180, and 187) were statistically significant, whereas the results for three congeners (28, 99, and 183) were not (Figure 2 and Table 4). All of the seven congeners with significant meta-analysis results have been previously described as having immunotoxic characteristics, and one (118) is also considered to be dioxin like.

### 3.2. Ecological Effects

**Figure 3:**
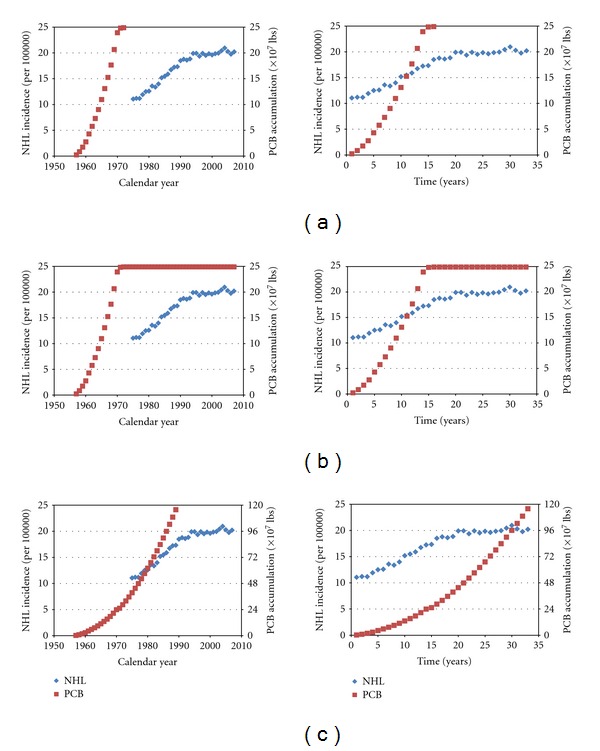
Ecological correlations between PCB accumulation and the national incidence of NHL compared over calendar years and relative year based on the assumptions of (a) sales-only exposure (sales model); (b) environmental exposure (static model); (c) physiologic exposure (dynamic model).

The ecological data representing the incidence of NHL versus PCB accumulation over time indicated statistically strong correlations (*R* ^2^ > 0.94) regardless of the assumed accumulation models (Figure 3). A high degree of correlation (*R* ^2^ > 0.8) between NHL incidence and PCB accumulation was observed for several other cancers for one or two of the accumulation models, including breast, liver and bile duct, kidney, skin, and soft tissue and heart, however none of these other cancers consistently demonstrated high-correlation values in all three models (Table 5).

## 4. Discussion

The results of the (nonexhaustive) application of the Hill criteria to the current state of knowledge regarding the relationship between blood levels of PCBs and NHL risk indicate a plausible causal association. The multiple case-control studies described herein also consistently demonstrate a strong level of association between elevated PCB levels and NHL sufficient to conclude that a (general) causal relationship exists between the two, and that if PCBs were to be eliminated from the environment, a certain proportion of NHL cases would likewise be eliminated.

 This conclusion is further supported by the results of the ecological analysis reported herein, in which a relationship between PCB levels in the environment over time appeared to be more uniquely and strongly correlated with NHL incidence than with other types of cancers. The multicause nature of NHL means that these data should be viewed with caution and even skepticism, but the observed relationships are at the very least consistent with other theories regarding the inordinately large increase in NHL incidence over the past approximately 40 years.

Based on these results and the multifactorial nature of NHL, it is reasonable to conclude that a certain proportion of individual cases of NHL occur *only* because of elevated PCB levels; in another proportion, elevated PCB levels have *contributed* to the cause of the NHL in conjunction with other causes but cannot be said to be solely necessary as a cause; in another proportion the body burden of PCBs is neither completely nor partially contributory to the NHL occurrence. We posit that if an individual with NHL is found to have a titer of PCB congeners 118, 138, 153, 156, 170, 180 or 187 that exceeds 75% of that of the comparable general population of the same age and era [75], then it can be concluded that the elevated body burden of PCBs causally contributed to the NHL occurrence.

Given this conclusion, the issue of the >2.0 relative risk specific causation threshold must be addressed. In a setting in which there is an identified unexposed comparison group, a relative risk of 1.43 (here defined by an OR with the lesser exposed) would mean that out of 143 hazard-exposed subjects with the disease of interest, 43 acquired the disease only because of the exposure, and 100 developed the disease independent of the exposure to the hazard. For the purposes of a specific causation evaluation, randomly selecting one of the exposed cases would result in a probability of causation of 30% (43/143), and thus the conclusion that the individual's disease was not related to the exposure, on a more probable than not basis. Such an approach is potentially problematic because it will result in an erroneous determination of no causal relationship between the hazardous exposure and the disease in 30% of specific causation evaluations [76]. The 2.0 relative risk approach becomes problematic to a point of impracticality when evaluating specific causation for PCBs and NHL. When considering the 143 highest PCB titer percentile-exposed subjects with NHL (representative of the meta-analyzed 1.43 OR_MH_ described herein), there will be 43 who have the disease only because of the PCB exposure, and 100 subjects in whom their elevated PCB levels may or may not have contributed to their NHL, since in reality all of the “unexposed” subjects are really just “lesser exposed” subjects. The fact that there are no unexposed comparison groups (zero PCB body burden) with which the cases could be compared in the meta-analyzed studies effectively lowered the resulting odds ratios to a largely unknown degree. Some indication of the magnitude of this effect can be inferred from the ecological data presented herein, as there was an approximate doubling of the incidence of NHL over a 30-year period of time that was temporally associated with the introduction of PCBs into the environment. It may be a reasonable supposition that some proportion of the cases resulted from the exponential increase in environmental PCBs that preceded the dramatic increase in the rate of NHL and which are represented in all percentiles of PCB exposure.

Additionally, it appears that there are a couple of factors that tend to decrease PCB levels in the body that may be associated with the presence or diagnosis of NHL, resulting in lower post-NHL-diagnosis titers than what may have been presented prior to diagnosis. Individuals with higher body mass index (BMI) levels metabolize and eliminate PCBs more slowly, and because weight loss is a common feature of NHL, this feature of the disease would tend to decrease the body burden of PCBs as the illness progressed [77]. Additionally, chemotherapy, a common medical treatment for NHL, has been observed to potentially decrease PCB levels in the body by nearly 30% [78].

Taken together, all of these factors indicate that the 2.0 relative risk or odds ratio threshold cannot be reasonably applied to PCB and NHL. It is for this reason that the use of upper percentile cutoff values associated with the maximum versus minimum OR_MH_ to conclude that causal contribution is present is thought to be a reasonably practicable alternative to evaluating causation for an environmental toxin so ubiquitous that no unexposed group exists for comparison.

## 5. Conclusions

 Application of the Hill criteria to the current state of knowledge regarding the association between environmental PCBs and NHL reveals convincing evidence of plausibility, as well as a strong general causal association, with meta-analyzed odds ratios indicating a 43% association of studied NHL cases in the literature with total PCB levels in the highest percentile, relative to comparison populations in the lowest percentiles of PCB levels. For evaluation of the causal contribution of PCBs to an individual case of NHL, the meta-analyzed values for seven immunotoxic congeners (118, 138, 153, 156, 170, 180, and 187) are presented and compared with relevant population survey data. When an individual case of NHL presents with one of these seven congener titers that fall into the highest quartile of their representative general population, it is reasonable to conclude a causal contributory relationship is present, on a more probable than not basis.

## Figures and Tables

**Table 1:** Summary of published case-control study populations examining risk of NHL and PCB blood content including those meta-analyzed in the present study. Summed or total PCB metrics shown here.

Study (notes, conc values)	Percentile	PCB conc (ng/g lipid)	Min conc	Max conc	Case sample size	Control sample size	OR	95% LCL	95% UCL	Meta-analysis inclusion
Rothman et al. 1997 (mean conc) [40]	Total				74	147				
	1	526	247	641	10	37	1.00			
	2	727	649	806	13	37	1.30	0.5	3.3	No
	3	924	814	1060	21	37	2.80	1.1	7.6	
	4	1430	1070	2070	30	36	4.50	1.7	12.0	

Engel et al., 2007 (Janus cohort, median conc) [41]	Total				190	190				
	1	1048.3			44	48	1.00			
	2	1398.3			48	47	1.10	0.7	2.0	Yes
	3	1674.9			38	48	1.00	0.5	1.9	
	4	2148.2			60	47	1.70	0.8	3.4	

Engel et al., 2007 (CLUE 1 cohort median conc) [41]	Total				74	147				
	1	551.4			10	37	1.00			
	2	726			13	37	1.10	0.7	2.0	Yes
	3	911.5			21	37	1.00	0.5	1.9	
	4	1377.3			30	36	1.70	0.8	3.4	

Spinelli et al., 2007 (Median conc) [42]	Total				403					
	1		<100.9	100.9	81	115	1.00			
	2		>100.9	155.6	103	114	1.41	0.93	2.14	Yes
	3		>155.6	220	77	115	1.11	0.71	1.74	
	4		>220	6571	142	115	2.14	1.38	3.30	

De Roos et al., 2005 [26]	Total				97	96				
	1		<6.4	6.4	28	24	1.00			
	2		>6.4	8.69	16	25	0.59	0.25	1.40	Yes
	3		>8.69	13.17	20	23	0.86	0.38	1.98	
	4		>13.17		33	24	1.51	0.62	3.67	

Laden et al., 2010 (median conc) [48]	Total				145	290				
	1	406.9			33	72	1.00			
	2	547.8			41	73	1.22	0.69	2.18	Yes
	3	678.0			41	73	1.22	0.69	2.18	
	4	945.4			30	72	0.91	0.50	1.67	

Hardell et al., 2001 (36 PCBs, mean conc) [53]	Total	1436	331	4706	82	83	1.00			
	1		1296 (med)	4706	51	41	1.80	0.85	3.9	No

Hardell et al., 2001 (11 immunotoxic PCBs) [53]	Total	511	118	1598	82	83	1.00			
	1		462 (med)	1598	57	40	3.20	1.4	7.4	No

Hardell et al., 2006; [54] Hardell et al., 2009 [43] (all PCBs, mean conc)	Total	860	102	4222	97	98	1.00			
	1		762 (med)	4222	59	49	2.00	0.99	3.99	No

Hardell et al., 2006; [54] Hardell et al., 2009 [43] (11 immunotoxic PCBs)	Total	290	41	1297	97	98	1.00			
	1		238 (med)	1297	54	49	1.50	0.8	3.0	No

Cocco et al., 2008 [46]	Total				174					
	1		<200.42	200.42	41	51	1.00			
	2		200.43	387.79	50	51	1.20	0.6	2.2	Yes
	3		387.8	576.36	33	50	0.70	0.3	1.4	
	4		576.37	>576.37	50	51	1.00	0.5	2.0	

Quintana et al., 2004 [44]	Total									
	1		<1000	1000	79	184	1.00			
	2		>1000	3000	50	151	1.05	0.63	1.76	No
	3		>3000		9	23	1.08	0.40	2.92	

Bertrand et al., 2010 (median conc) [47]	Total				205	409				
	1	518	163	617	33	81	1..00			
	2	678	>617	742	31	82	0.92	0.51	1.60	Yes
	3	815	>742	894	34	82	1.00	0.60	1.80	
	4	980	>894	1121	46	82	1.40	0.71	2.20	
	5	1385	>1121	5322	61	82	1.90	0.94	2.90	

**Table 2:** Summary of published cohort studies examining mortality from NHL as associated with PCB exposure. Note the cohort described in Prince et al., [50] were included in the Prince et al., [51] study. Here, the standardized mortality ratio (SMR) was calculated as an indirect adjustment method where the observed number of events (deaths) in each occupational cohort is compared with the number of expected events (based on a “standard” rate).

Study	PCB product exposure	Chlorine (%)	PCB air sampling (*μ*g/m^3^)	Cohort size (*n*)	Deaths	Cancer deaths	NHL deaths	Mortality rate (SMR)
Prince et al., 2006 [50]	Aroclor 1254	54	24–476 & 50–1,260	2,572	798	218	10	1.31
	Aroclor 1242	42						
	Aroclor 1016	41						

Prince et al., 2006 [51]	Aroclor 1254	54	24–476 & 50–1,260	14,458	3,417	1015	35	0.98
	Aroclor 1242	42						
	Aroclor 1016	41						

Ruder et al., 2006 [52]	Aroclor 1242	42	7–339 & 62–290	3,569	547	171	9	1.23
	Aroclor 1016	41						

**Table 3:** Notation for the calculation of Mantel-Haenszel-adjusted odds ratios from the published case-control studies reporting association of NHL and PCB blood content for specific congeners. Here, “exposed” and “unexposed” populations noted in traditional meta-analyses were replaced with “upper” and “lower” acknowledging the upper and lower percentiles of exposure reported in the literature, respectively. The subscript “*i*” indicates each stratum (published congener).

	Cases	Controls	Total
Upper	*a*_*i*_	*b*_*i*_	*M*_1*i*_
Lower	*c*_*i*_	*d*_*i*_	*M*_2*i*_
Total	*n*_1*i*_	*n*_2*i*_	*N*_*i*_

**Table 4:** List of contributing strata and OR metrics satisfying the meta-analysis criteria. Results of the Chi-square test for homogeneity (*P* values and degrees of freedom, DOF) are shown for each meta-analyzed congener. Note that congener 118 is statistically heterogeneous (*P* < 0.025). However, removal of the weighted outlier [46] indicated homogeneity in the remaining strata. A combined test of all congeners indicated overall homogeneity (*P* > 0.10 and 37 DOF).

Study (strata)	Percentile type	PCB congener	OR	LCL	UCL	Homogeneity test (Chi-Square DOF)
Spinelli et al., 2007 [42]	Tertile	28	0.95	0.67	1.34	*P* > 0.10 (1)
Cocco et al., 2008 [46]	Quartile	28	1.60	0.80	3.20	

Spinelli et al., 2007 [42]	Quartile	99	1.27	0.86	1.87	*P* > 0.10 (1)
De Roos et al., 2005 [26]	Quartile	99	0.77	0.28	2.10	

Engel et al., 2007-J [41]	Quartile	118	1.70	0.90	3.50	*P* < 0.025 (6)
Engel et al., 2007-C [41]	Quartile	118	5.40	1.70	17.10	
Spinelli et al., 2007 [42]	Quartile	118	1.77	1.15	2.72	
De Roos et al., 2005 [26]	Quartile	118	0.73	0.29	1.84	
Cocco et al., 2008 [46]	Quartile	118	0.40	0.20	0.80	
Laden et al., 2010 [48]	Quartile	118	0.81	0.42	1.56	
Bertrand et al., 2010 [47]	Quintile	118	1.40	0.76	2.50	

Engel et al., 2007-J [41]	Quartile	138	1.70	0.80	3.20	*P* > 0.10 (5)
Engel et al., 2007-C [41]	Quartile	138	4.40	1.50	12.60	
Spinelli et al., 2007 [42]	Quartile	138	1.46	0.98	2.18	
Cocco et al., 2008 [46]	Quartile	138	1.10	0.60	2.00	
Laden et al., 2010 [48]	Quartile	138	0.95	0.49	1.83	
Bertrand et al., 2010 [47]	Quintile	138	1.80	0.98	3.20	

Engel et al., 2007-J [41]	Quartile	153	2.00	1.00	3.90	*P* > 0.10 (6)
Engel et al., 2007-C [41]	Quartile	153	2.20	0.90	5.20	
Spinelli et al., 2007 [42]	Quartile	153	1.79	1.17	2.72	
De Roos et al., 2005 [26]	Quartile	153	1.59	0.63	4.00	
Cocco et al., 2008 [46]	Quartile	153	1.00	0.70	2.50	
Laden et al., 2010 [48]	Quartile	153	0.82	0.43	1.56	
Bertrand et al., 2010 [47]	Quintile	153	2.10	1.10	3.80	

Spinelli et al., 2007 [42]	Quartile	156	1.77	1.14	2.74	*P* > 0.10 (1)
De Roos et al., 2005 [26]	Quartile	156	2.70	0.97	7.50	

Spinelli et al., 2007 [42]	Quartile	170	1.80	1.16	2.79	*P* > 0.10 (2)
De Roos et al., 2005 [26]	Quartile	170	1.73	0.73	4.14	
Cocco et al., 2008 [46]	Quartile	170	1.00	0.50	1.80	

Spinelli et al., 2007 [42]	Quartile	180	1.91	1.19	3.07	*P* > 0.10 (4)
De Roos et al., 2005 [26]	Quartile	180	3.50	1.34	9.15	
Laden et al., 2010 [48]	Quartile	180	1.03	0.52	2.02	
Cocco et al., 2008 [46]	Quartile	180	1.50	0.70	3.20	
Bertrand et al., 2010 [47]	Quintile	180	2.40	1.30	4.50	

Spinelli et al., 2007 [42]	Tertile	183	1.22	0.87	1.71	*P* > 0.10 (1)
De Roos et al., 2005 [26]	Quartile	183	1.02	0.36	2.93	

Spinelli et al., 2007 [42]	Quartile	187	1.92	1.23	2.98	*P* > 0.10 (1)
De Roos et al., 2005 [26]	Quartile	187	1.22	0.49	3.08	

**Table 5:** Summary of the dose-response correlations between synchronized polychlorinated biphenyl (PCB) bioaccumulation and the incidence of cancer. Accumulation is represented as based on sales alone (sales growth), environmental exposure (static growth), and physiologic exposure (dynamic growth).

Cancer type	Statistical correlation with PCB accumulation (*R* ^2^)		
	Sales only (sales)	Environment (static)	Physiologic (dynamic)
Non-Hodgkin lymphoma	0.9813	0.9427	0.9850
All cancers	0.9754	0.8874	0.3523
Hodgkin lymphoma	0.2077	0.0062	0.0273
Pancreatic	0.0411	0.0979	0.0001
Liver and bile duct	0.8834	0.5960	0.9715
Kidney and renal	0.9399	0.7022	0.9534
Bone and joint	0.1190	0.1156	0.1486
Brain and nervous system	0.7966	0.5746	0.0800
Digestive system	0.0089	0.4168	0.8548
Leukemia	0.0913	0.0248	0.0081
Lung	0.8826	0.5600	0.0236
Skin	0.9166	0.7254	0.9378
Oral	0.4224	0.5994	0.8910
Soft tissue and heart	0.3322	0.5377	0.9118
Urinary bladder	0.7153	0.7050	0.3750
Breast (female)	0.9076	0.8639	0.3546
Genital system (female)	0.6892	0.7718	0.6825
Prostate (male)	0.8369	0.7290	0.3808

## References

[1] Agency for Toxic Substances and Disease Registry (ATSDR) (2000). *Toxicological Profile for Polychlorinated Biphenyls (PCBs)*.

[2] National Toxicology Program (NTP) (2004). *Report on Carcinogens, Substance Profiles*.

[3] Environmental Protection Agency (EPA) *Polychlorinated Biphenyls (PCBs)*.

[4] Environmental Protection Agency (EPA) Health assessment document for 2,3,7,8-tetrachlorodibenzo-p-dioxin (TCDD) and related compounds. Prepared by the Office of Health and Environmental Assessment, Office of Research and Development, Washington, DC. External Review Draft, 3 vol.

[5] Safe S (1990). Polychlorinated biphenyls (PCBs), dibenzo-p-dioxins (PCDDs), dibenzofurans (PCDFs), and related compounds: environmental and mechanistic considerations which support the development of toxic equivalency factors (TEFs). *Critical Reviews in Toxicology*.

[6] Monsanto, Polychlorinated Biphenyls (PCBs) (1979). *A Report on Uses, Environmental and Health Effects and Disposal*.

[7] Cogliano VJ (1998). Assessing the cancer risk from environmental PCBs. *Environmental Health Perspectives*.

[8] Johnson BL, Hicks HE, Cibulas W (1999). *Public Health Implications of Exposure to Polychlorinated Biphenyls (PCBs)*.

[9] Safe S (1992). Toxicology, structure-function relationship, and human and environmental health impacts of polychlorinated biphenyls: progress and problems. *Environmental Health Perspectives*.

[10] Hanrahan LP, Falk C, Anderson HA (1999). Serum PCB and DDE levels of frequent Great Lakes sport fish consumers—a first look. *Environmental Research A*.

[11] McKinney JD, Waller CL (1994). Polychlorinated biphenyls as hormonally active structural analogues. *Environmental Health Perspectives*.

[12] Environmental Protection Agency (EPA) (1996). PCBs: cancer dose-response assessment and application to environmental mixtures.

[13] Kimbrough RD, Squire RA, Linder RE, Strandberg JD, Montalli RJ, Burse VW (1975). Induction of liver tumors in Sherman strain female rats by polychlorinated biphenyl aroclor 1260. *Journal of the National Cancer Institute*.

[14] Norback DH, Weltman RH (1985). Polychlorinated biphenyl induction of hepatocellular carcinoma in the Sprague-Dawley rat. *Environmental Health Perspectives*.

[15] Schaeffer E, Greim H, Goessner W (1984). Pathology of chronic polychlorinated biphenyl (PCB) feeding in rats. *Toxicology and Applied Pharmacology*.

[16] Brunner MJ, Sullivan TM, Singer AW (1996). An assessment of the chronic toxicity and oncogenicity of Aroclor-1016, Aroclor-1242, Aroclor-1254, and Aroclor-1260 administered in diet to rats. *Study no.*.

[17] Brown DP (1987). Mortality of workers exposed to polychlorinated biphenyls—an update. *Archives of Environmental Health*.

[18] Recio-Vega R, Velazco-Rodriguez V, Ocampo-Gómez G, Hernandez-Gonzalez S, Ruiz-Flores P, Lopez-Marquez F (2011). Serum levels of polychlorinated biphenyls in Mexican women and breast cancer risk. *Journal of Applied Toxicology*.

[19] Gallagher RP, Macarthur AC, Lee TK (2011). Plasma levels of polychlorinated biphenyls and risk of cutaneous malignant melanoma: a preliminary study. *International Journal of Cancer*.

[20] Maifredi G, Donato F, Magoni M (2011). Polychlorinated biphenyls and non-Hodgkin's lymphoma: a case-control study in Northern Italy. *Environmental Research*.

[21] Schantz SL (1996). Developmental neurotoxicity of PCBs in humans: what do we know and where do we go from here?. *Neurotoxicology and Teratology*.

[22] Levin M, Morsey B, Mori C, Nambiar PR, De Guise S (2005). Non-coplanar PCB-mediated modulation of human leukocyte phagocytosis: a new mechanism for immunotoxicity. *Journal of Toxicology and Environmental Health A*.

[23] Selgrade MK (2007). Immunotoxicity—the risk is real. *Toxicological Sciences*.

[24] Grulich AE, Vajdic CM, Cozen W (2007). Altered immunity as a risk factor for non-Hodgkin lymphoma. *Cancer Epidemiology Biomarkers and Prevention*.

[25] Engels EA, Cerhan JR, Linet MS (2005). Immune-related conditions and immune-modulating medications as risk factors for non-Hodgkin’s lymphoma: a case-control study. *American Journal of Epidemiology*.

[26] De Roos AJ, Hartge P, Lubin JH (2005). Persistent organochlorine chemicals in plasma and risk of non-Hodgkin’s lymphoma. *Cancer Research*.

[27] Merhi M, Raynal H, Cahuzac E, Vinson F, Cravedi JP, Gamet-Payrastre L (2007). Occupational exposure to pesticides and risk of hematopoietic cancers: meta-analysis of case-control studies. *Cancer Causes and Control*.

[28] Marieb EN (2001). *Human Anatomy and Physiology*.

[29] Ferlay J, Bray F, Pisani P, Parkin DM (2001). *GLOBOCAN 2000: Cancer Incidence, Mortality and Prevalence Worldwide*.

[30] Melbye M, Trichopoulos D, Adami HO, Hunter D, Trichopoulos D (2002). Non-Hodgkin's lymphomas. *Textbook of Cancer Epidemiology*.

[31] Ries LAG, Melbert D, Krapcho M SEER cancer statistics review, 1975–2005. http://seer.cancer.gov/csr/1975_2005/.

[32] Altekruse SF, Kosary CL, Krapcho M SEER cancer statistics review, 1975–2007. http://seer.cancer.gov/csr/1975_2007/.

[33] Hardell L (2008). Pesticides, soft-tissue sarcoma and non-Hodgkin lymphoma—historical aspects on the precautionary principle in cancer prevention. *Acta Oncologica*.

[34] Hardell L, Eriksson M (2003). Is the decline of the increasing incidence of non-Hodgkin lymphoma in Sweden and other countries a result of cancer preventive measures. *Environmental Health Perspectives*.

[35] Mueller NE, Mohar A, Evans A (1992). Viruses other than HIV and non-Hodgkin’s lymphoma. *Cancer Research*.

[36] Fisher SG, Fisher RI (2004). The epidemiology of non-Hodgkin’s lymphoma. *Oncogene*.

[37] Kafafi SA, Afeefy HY, Ali AH, Said HK, Kafafi GA (1993). Binding of polychlorinated biphenyls to the aryl hydrocarbon receptor. *Environmental Health Perspectives*.

[38] Tijet N, Boutros PC, Moffat ID, Okey AB, Tuomisto J, Pohjanvirta R (2006). Aryl hydrocarbon receptor regulates distinct dioxin-dependent and dioxin-independent gene batteries. *Molecular Pharmacology*.

[39] Rocca CL, Alivernini S, Badiali M (2008). TEQS and body burden for PCDDs, PCDFs, and dioxin-like PCBs in human adipose tissue. *Chemosphere*.

[40] Rothman N, Cantor KP, Blair A (1997). A nested case-control study of non-Hodgkin lymphoma and serum organochlorine residues. *The Lancet*.

[41] Engel LS, Laden F, Andersen A (2007). Polychlorinated biphenyl levels in peripheral blood and non-Hodgkin’s lymphoma: a report from three cohorts. *Cancer Research*.

[42] Spinelli JJ, Ng CH, Weber JP (2007). Organochlorines and risk of non-Hodgkin lymphoma. *International Journal of Cancer*.

[43] Hardell L, Eriksson M, Lindström G (2001). Case-control study on concentrations of organohalogen compounds and titers of antibodies to Epstein-Barr virus antigens in the etiology of non-Hodgkin lymphoma. *Leukemia and Lymphoma*.

[44] Hardell L, Bjornfoth H, Hardell K, van Bavel B, Lindstrom G, Carlberg M (2006). Concentrations of organohalogen compounds and titers of antibodies to Epstein-Barr virus antigens and the risk for non-Hodgkin lymphoma. *Organohalogen Compounds*.

[45] Hardell K, Carlberg M, Hardell L (2009). Concentrations of organohalogen compounds and titres of antibodies to Epstein-Barr virus antigens and the risk for non-Hodgkin lymphoma. *Oncology Reports*.

[46] Cocco P, Brennan P, Ibba A (2008). Plasma polychlorobiphenyl and organochlorine pesticide level and risk of major lymphoma subtypes. *Occupational and Environmental Medicine*.

[47] Bertrand KA, Spiegelman D, Aster JC (2010). Plasma organochlorine levels and risk of non-hodgkin lymphoma in a cohort of men. *Epidemiology*.

[48] Laden F, Bertrand KA, Altshul L, Aster JC, Korrick SA, Sagiv SK (2010). Plasma organochlorine levels and risk of non-Hodgkin lymphoma in the nurses' health study. *Cancer Epidemiology Biomarkers and Prevention*.

[49] Quintana PJE, Delfino RJ, Korrick S (2004). Adipose tissue levels of organochlorine pesticides and polychlorinated biphenyls and risk of non-Hodgkin’s lymphoma. *Environmental Health Perspectives*.

[50] Prince MM, Hein MJ, Ruder AM, Waters MA, Laber PA, Whelan EA (2006). Update: cohort mortality study of workers highly exposed to polychlorinated biphenyls (PCBs) during the manufacture of electrical capacitors, 1940–1998. *Environmental Health*.

[51] Prince MM, Ruder AM, Hein MJ (2006). Mortality and exposure response among 14,458 electrical capacitor manufacturing workers exposed to polychlorinated biphenyls (PCBs). *Environmental Health Perspectives*.

[52] Ruder AM, Hein MJ, Nilsen N (2006). Mortality among workers exposed to polychlorinated biphenyls (PCBs) in an electrical capacitor manufacturing plant in Indiana: an update. *Environmental Health Perspectives*.

[53] Colt JS, Severson RK, Lubin J (2005). Organochlorines in carpet dust and non-Hodgkin lymphoma. *Epidemiology*.

[54] Morton LM, Wang SS, Cozen W (2008). Etiologic heterogeneity among non-Hodgkin lymphoma subtypes. *Blood*.

[55] Freeman MD, Kohles SS (2011). An evaluation of applied biomechanics as an adjunct to systematic specific causation in forensic medicine. *Wiener Medizinische Wochenschrift*.

[56] Freeman MD, Kohles SS (2011). Application of the Hill criteria to the causal association of post-traumatic headache with assault. *Egyptian Journal of Forensic Science*.

[57] Loue S (2010). *Forensic Epidemiology: Integrating Public Health and Law Enforcement*.

[59] Department of Health and Human Services (DHHS) (2000). *Guidelines for Determining the Probability of Causation and Methods for Radiation Dose Reconstruction Under the Employees Occupational Illness Compensation Program Act of 2000*.

[60] Smith FM (2000). *Reference Manual on Scientific Evidence*.

[61] Rothman KJ, Greenland S (2005). Causation and causal inference in epidemiology. *American Journal of Public Health*.

[62] Hill AB (1965). The environment and disease: association or causation?. *Proceedings of the Royal Society of Medicine*.

[63] van Reekum R, Streiner DL, Conn DK (2001). Applying Bradford Hill’s criteria for causation to neuropsychiatry: challenges and opportunities. *Journal of Neuropsychiatry and Clinical Neurosciences*.

[64] Lozano-Calderón S, Anthony S, Ring D (2008). The quality and strength of evidence for etiology: example of carpal tunnel syndrome. *Journal of Hand Surgery*.

[65] Batty L, Holland-Elliott K, Rosenfeld D (2003). Investigation of eye splash and needlestick incidents from an HIV-positive donor on an intensive care unit using root cause analysis. *Occupational Medicine*.

[66] Freeman MD, Centeno CJ, Kohles SS (2009). A systematic approach to clinical determinations of causation in symptomatic spinal disk injury following motor vehicle crash trauma. *PM & R*.

[67] Thygesen LC, Andersen GS, Andersen H (2005). A philosophical analysis of the Hill criteria. *Journal of Epidemiology and Community Health*.

[68] Rothman KJ, Greenland S (1998). *Modern Epidemiology*.

[69] Robins J, Greenland S, Breslow NE (1986). A general estimator for the variance of the Mantel-Haenszel odds ratio. *American Journal of Epidemiology*.

[70] Pagano M, Gauvreau K (2000). *Principles of Biostatistics*.

[71] Vinson F, Merhi M, Baldi I, Raynal H, Gamet-Payrastre L (2011). Exposure to pesticides and risk of childhood cancer: a meta-analysis of recent epidemiological studies. *Occupational and Environmental Medicine*.

[72] DerSimonian R, Laird N (1986). Meta-analysis in clinical trials. *Controlled Clinical Trials*.

[73] Interdepartmental Task Force on PCBs (1972). *Polychlorinated Biphenyls and the Environment*.

[74] Wolff MS, Camann D, Gammon M, Stellman SD (1997). Proposed PCB congener groupings for epidemiological studies. *Environmental Health Perspectives*.

[75] National Health and Nutrition Examination Survey (NHANES) (2011). *Centers for Disease Control and Prevention (CDC)*.

[76] Golanski A (2003). General causation at a crossroads in toxic tort cases. *Penn State Law Review*.

[77] Wolff MS, Britton JA, Teitelbaum SL (2005). Improving organochlorine biomarker models for cancer research. *Cancer Epidemiology Biomarkers and Prevention*.

[78] Baris D, Kwak LW, Rothman N (2000). Blood levels of organochlorines before and after chemotherapy among non-Hodgkin’s lymphoma patients. *Cancer Epidemiology Biomarkers and Prevention*.

